# Fucosterol, a Phytosterol of Marine Algae, Attenuates Immobilization-Induced Skeletal Muscle Atrophy in C57BL/6J Mice

**DOI:** 10.3390/md22120557

**Published:** 2024-12-12

**Authors:** Jieun Hwang, Mi-Bo Kim, Sanggil Lee, Jae-Kwan Hwang

**Affiliations:** 1 Department of Biotechnology, College of Life Science and Biotechnology, Yonsei University, Seoul 03722, Republic of Korea; 19jieun92@hanmail.net (J.H.); mibokim1120@gmail.com (M.-B.K.); 2 Department of Food Science and Nutrition, Pukyong National University, Busan 48513, Republic of Korea; 3 Department of Smart Green Technology Engineering, Pukyong National University, Busan 48513, Republic of Korea

**Keywords:** fucosterol, skeletal muscle atrophy, immobilization, Akt/mTOR/FoxO3α pathway

## Abstract

The objective of this study was to examine whether fucosterol, a phytosterol of marine algae, could ameliorate skeletal muscle atrophy in tumor necrosis factor-alpha (TNF-α)-treated C2C12 myotubes and in immobilization-induced C57BL/6J mice. Male C57BL6J mice were immobilized for 1 week to induce skeletal muscle atrophy. Following immobilization, the mice were administrated orally with saline or fucosterol (10 or 30 mg/kg/day) for 1 week. Fucosterol significantly attenuated immobilization-induced muscle atrophy by enhancing muscle strength, with a concomitant increase in muscle volume, mass, and myofiber cross-sectional area in the tibialis anterior (TA) muscle in mice. In both the TNF-α-treated C2C12 myotubes and the TA muscle of immobilized mice, fucosterol significantly prevented muscle protein degradation, which was attributed to a reduction in atrogin-1 and muscle ring finger 1 gene expression through an increase in forkhead box O3α (FoxO3α) phosphorylation. Continuously, fucosterol stimulated muscle protein synthesis by increasing the phosphorylation of the mammalian target of the rapamycin (mTOR), 70 kDa ribosomal protein S6 kinase, and 4E binding protein 1, which was mediated through the stimulation of the phosphatidylinositol 3-kinase (PI3K)/Akt signaling pathway. Thus, fucosterol alleviated skeletal muscle atrophy in TNF-α-treated C2C12 myotubes and immobilized C57BL/6J mice through the regulation of the Akt/mTOR/FoxO3α signaling pathway.

## 1. Introduction

Skeletal muscle constitutes 40% of human body weight, playing a vital role in maintaining physiological metabolism, enabling physical activity and storing crucial substances such as glucose and amino acids [1]. Skeletal muscle mass represents an important determinant of its physiological functions, encompassing strength and exercise capacity [2]. However, skeletal muscle atrophy is a critical issue, marked by the loss of skeletal muscle mass, and it histologically reveals a reduction in both the quantity and size of myofibers [3]. The loss of skeletal muscle mass can occur due to immobilization (disuse), aging (sarcopenia), and chronic diseases (cachexia) such as diabetes, cancer, and advanced immune deficiency syndrome [4]. Furthermore, skeletal muscle atrophy precipitates a significant reduction in physical performance and mobility, consequently leading to a diminished quality of life and life expectancy. Therefore, skeletal muscle atrophy is a significant public health concern that can reduce health-related quality of life [5].

Several studies have demonstrated that patients suffering from disease-related skeletal muscle atrophy have elevated levels of circulating endotoxin and persistent systemic inflammation, suggesting that inflammation plays an important role in the pathogenesis of skeletal muscle atrophy [6]. In particular, tumor necrosis factor-alpha (TNF-α) is recognized as a major pro-inflammatory cytokine involved in local and systemic inflammation [4]. In the skeletal muscle, TNF-α disrupts the anabolism of muscle proteins by inhibiting the mammalian target of rapamycin (mTOR) signaling pathway, while simultaneously promoting the catabolism of muscle proteins through the activation of the ubiquitin–proteasome system [4,7]. Ultimately, systemic inflammation in the skeletal muscle accelerates the breakdown of muscle proteins, resulting in muscle atrophy. Thus, it is necessary to find natural bioactive components that potently inhibit inflammatory cytokine properties, which could be represent an attractive resource in preventing skeletal muscle mass loss during muscle atrophy.

**Figure 1:**
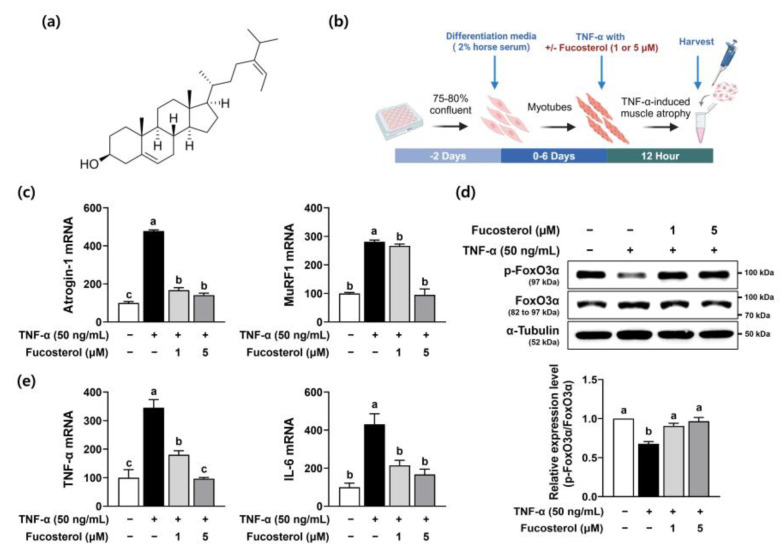
Inhibitory effects of fucosterol on muscle protein degradation-related markers in TNF-α-induced C2C12 myotubes. (**a**) Structure of fucosterol. (**b**) Experimental design. After 6 days of differentiation, C2C12 myotubes were treated with TNF-α (50 ng/mL) and fucosterol (1 and 5 µM) for 12 h. (**c**) The gene expression of atrogin-1 and MuRF1 was analyzed by RT-PCR. (**d**) The protein expression of FoxO3α phosphorylation was analyzed by Western blotting. (**e**) The gene expression of TNF-α and IL-6 was analyzed by RT-PCR. β-Actin and α-tubulin was utilized as the internal control in RT-PCR and Western blot analyses, respectively. The data displayed represent the mean values accompanied by the standard deviation. The bars labeled with different letters indicate statistically significant differences between the groups (*p* < 0.05).

Fucosterol (Figure 1a), a phytosterol, is abundant in edible brown algae. Studies have demonstrated that fucosterol possesses anti-diabetes, anti-cancer, and antioxidant properties [8]. Notably, fucosterol has been documented to be a robust anti-inflammatory phytosterol that inhibits inducible nitric oxide synthase and pro-inflammatory cytokine secretion through the suppression of both the nuclear factor-kappa B (NF-κB) and p38 mitogen-activated protein kinase (MAPK) signaling pathways in lipopolysaccharide-treated macrophages [8,9,10]. However, whether fucosterol has anti-muscle atrophy properties in vitro or in vivo has not yet been determined. In the present study, we examined the ameliorative effects of fucosterol on TNF-α-induced muscle atrophy in C2C12 myotubes and immobilization-induced atrophied muscle in C57BL/6J mice to gain insights into its potential role in the development of muscle atrophy. Furthermore, our study reveals the first evidence that fucosterol exerts anti-muscle atrophy effects by inhibiting E3 ubiquitin ligases and activating the Akt/mTOR/forkhead box O3a (FoxO3α) signaling pathways in the TNF-α-treated C2C12 myotubes and immobilized hindlimb muscles of C57BL/6J mice.

## 2. Results

### 2.1. Fucosterol Attenuated TNF-α-Induced C2C12 Myotube Muscle Atrophy

**Figure 2:**
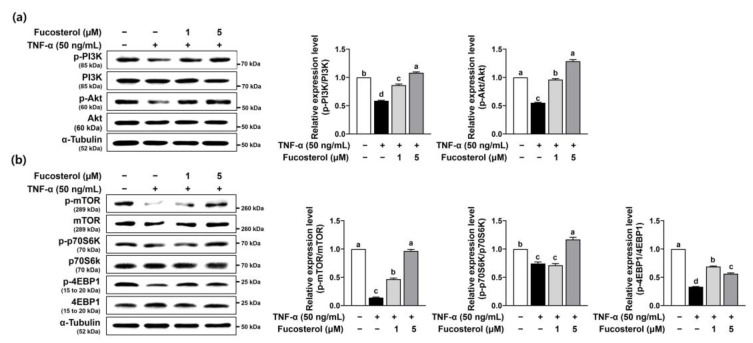
Enhancement effects of fucosterol on muscle protein synthesis-related markers in TNF-α-induced C2C12 myotubes. C2C12 myotubes were treated with TNF-α (50 ng/mL) and fucosterol (1 and 5 µM) for 12 h. (**a**,**b**) The protein expression of PI3K, Akt, mTOR, and p70S6K, and 4EBP1 phosphorylation, were analyzed by Western blotting. α-Tubulin was utilized as the internal control in Western blot analysis. The data displayed represent the mean values accompanied by the standard deviation. Bars labeled with different letters indicate statistically significant differences between groups (*p* < 0.05).

The pro-inflammatory cytokine TNF-α is recognized as a key factor responsible for the degradation of myofibrillar proteins via the ubiquitin–proteasome signaling pathway in the progression of skeletal muscle wasting diseases [11]. Thus, to examine the preventive effect of fucosterol in skeletal muscle atrophy, C2C12 myotubes were exposed to TNF-α to stimulate muscle atrophy. TNF-α significantly stimulated the gene expression of muscle-specific E3 ubiquitin ligases, such as atrogin-1 and muscle RING-finger 1 (MuRF1) in C2C12 myotubes, which was significantly diminished by fucosterol (Figure 1b). These muscle-specific E3 ubiquitin ligases are triggered into action by the transcription factor FoxO3α, which is essential in regulating protein degradation [12]. The protein expression of phosphorylated FoxO3α was significantly reduced by TNF-α, which was noticeably restored by fucosterol in C2C12 myotubes (Figure 1c). Furthermore, the gene expression levels of inflammatory cytokines, such as TNF-α and interleukin-6 (IL-6), were significantly elevated by TNF-α stimulation, which was dose-dependently inhibited by fucosterol (Figure 1d). The phosphatidylinositol 3-kinase (PI3K)/Akt signaling pathway is recognized as the primary trigger for the mTOR/P70S6K/4E-BP1 pathway, which is essential in driving protein synthesis [13]. In C2C12 myotubes, the phosphorylation of PI3K and Akt by TNF-α was reduced by TNF-α stimulation, but fucosterol noticeably recovered protein levels in phosphorylated PI3K and Akt (Figure 2a). Also, TNF-α markedly reduced the protein expression of protein synthesis markers in phosphorylated mTOR, 70 kDa ribosomal protein S6 kinase (p70S6K), and 4E binding protein 1 (4EBP1), which were markedly increased by fucosterol (Figure 2b).

### 2.2. Fucosterol Inhibited Immobilization-Induced Muscle Atrophy in C57BL/6J Mice

**Figure 3:**
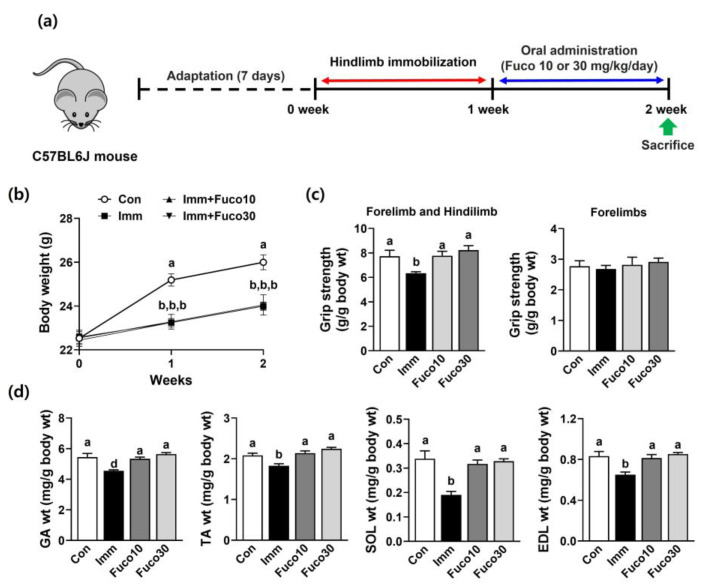
Effect of fucosterol on grip strength and muscle weights in immobilization-induced muscle atrophy in C57BL/6J mice. (**a**) Experimental design. After 1 week of muscle atrophy caused by immobilization, mice were subjected to the oral administration of saline or fucosterol (10 or 30 mg/kg/day) for 1 week. (**b**) Body weight. (**c**) The grip strength of the forelimb and hindlimb combined (four paws), as well as the forelimb alone (two paws). (**d**) Weights of different types of muscles, such as GA, SOL, TA, and EDL. *n* = 7 per group. The data displayed represent the mean values accompanied by the standard deviation. Groups denoted by distinct lettering exhibit statistically significant differences from each other (*p* < 0.05).

**Figure 4:**
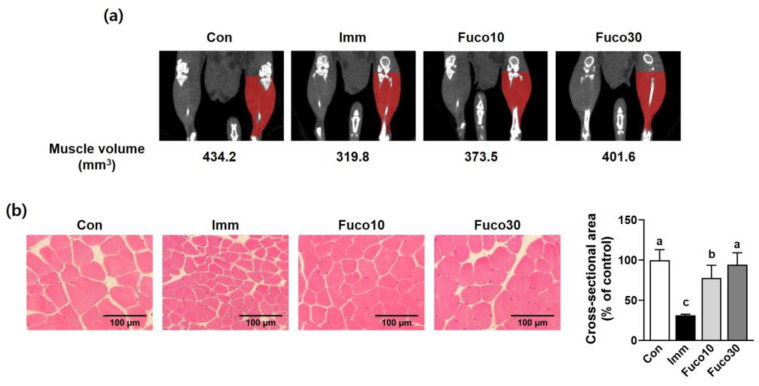
Effect of fucosterol on muscle volume and CSA in immobilization-induced muscle atrophy in C57BL/6J mice. After 1 week of muscle atrophy caused by immobilization, mice were subjected to the oral administration of saline or fucosterol (10 or 30 mg/kg/day) for 1 week. (**a**) Representative images of micro-CT. *n* = 1 per group. (**b**) Representative images of TA muscle sections stained with hematoxylin and eosin (H&E) for myofiber size and quantification in the CSA (% of control). *n* = 7 per group. The data displayed represent the mean values accompanied by the standard deviation. Groups denoted by distinct lettering exhibit statistically significant differences from each other (*p* < 0.05).

Studies have shown that an intake of fucosterol in the range of 20–300 mg/kg/day has beneficial effects [8]. Also, fucosterol is found in brown seaweed at concentrations of 8.2 to 32.2 mg/g dry weight [14]. Therefore, we investigated whether administering fucosterol at dosages of 10 and 30 mg/kg/day could attenuate muscle atrophy induced by immobilization in mice (Figure 3a). Immobilization stress exposure for 1 week decreased body weight between the Con group and the Imm group, including the Fuco10 and Fuco30 groups. However, there was no significant difference in body weight between the Imm group and the fucosterol administration groups (Fuco10 and Fuco 30) (Figure 3b). To evaluate whether fucosterol could increase muscle function in mice, a grip strength test was carried out to evaluate muscular force. The immobilization-induced skeletal muscle atrophy (Imm) group exhibited a significant decrease in grip strength in the combined forelimb and hindlimb compared to the Con group. However, fucosterol administration significantly mitigated the reduction in grip strength. There was no significant difference in forelimb grip strength between the groups (Figure 3c). In the Imm group, gastrocnemius (GA) muscle weight was significantly reduced compared to the Con group, but it was significantly increased by fucosterol administration in the Fuco10 and Fuco30 groups (Figure 3d). Also, the weight of other types of muscles, such as the tibialis anterior (TA), soleus (SOL), and extensor digitorum longus (EDL), was reduced by immobilization and was recovered by fucosterol administration, similarly to the Con group. Micro-computed tomography (micro-CT) results consistently showed more severe skeletal muscle atrophy in the Imm group due to the reduced muscle volume compared to the Con group, but this was attenuated by fucosterol administration (Figure 4a). Increasing the cross-sectional area (CSA) of skeletal muscle is known to enhance its ability to generate force through a tendon during physical activity, thereby resulting in significantly improved muscle strength [15]. Thus, we analyzed TA muscle tissue histologically to determine whether the increase in muscle strength resulting from fucosterol administration could be attributed to an increase in CSA. CSA in the TA muscle section in the Imm group showed a reduction of 68.7% compared to the Con group, but the Fuco10 and Fuco30 groups exhibited a significant increase in muscle section CSA by fucosterol administration (Figure 4b).

### 2.3. Fucosterol Inhibited the Expression of Pro-Inflammatory Cytokines and Protein Degradation-Related Markers in TA Muscle in Immobilized Mice

**Figure 5:**
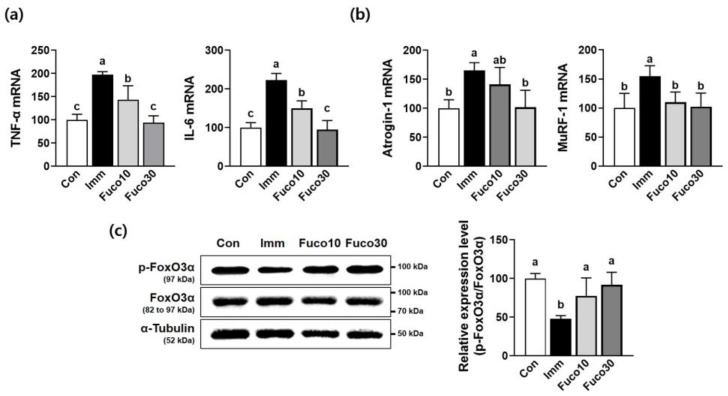
Inhibitory effect of fucosterol on the expression of pro-inflammatory cytokine expression and E3 ubiquitin ligases in TA muscle in immobilized-induced muscle atrophy C57BL/6J mice. After 1 week of muscle atrophy caused by immobilization, mice were subjected to the oral administration of saline or fucosterol (10 or 30 mg/kg/day) for 1 week. (**a**) The gene expression of TNF-α and IL-6 was analyzed in TA muscle by RT-PCR. (**b**) The gene expression of MuRF1 and atrogin-1 was analyzed in TA muscle by RT-PCR. (**c**) The protein expression of FoxO3α phosphorylation was analyzed in TA muscle by Western blotting. *n* = 3–5 per group. β-Actin and α-tubulin were utilized as the internal control in RT-PCR and Western blot analyses, respectively. The data displayed represent the mean values accompanied by the standard deviation. Groups denoted by distinct lettering exhibit statistically significant differences from each other (*p* < 0.05).

Immobilization-induced muscle atrophy is characterized by the increased production of pro-inflammatory cytokines, along with the upregulation of muscle-specific E3 ubiquitin ligases [16]. Thus, as we observed that fucosterol attenuated immobilization-induced muscle atrophy, concomitantly enhancing muscle function and mass in mice, we next evaluated the expression of pro-inflammatory cytokines and protein degradation-related markers in the TA muscles of mice. The Imm group showed a higher expression of pro-inflammatory cytokines, such as TNF-α and IL-6, than the Con group. However, this expression was significantly suppressed by fucosterol administration in the Fuco10 and Fuco30 groups (Figure 5a). Continuously, the expression of E3 ubiquitin ligase genes, including atrogin-1 and MuRF1, was significantly increased by immobilization in the Imm group, which was attenuated by fucosterol administration (Figure 5b). Also, the expression of phosphorylation of FoxO3α protein was significantly reduced by immobilization stress in the Imm group compared to the Con group, but the Fuco10 and Fuco30 groups showed an increase in the phosphorylation of FoxO3α protein expression at a similar level to the Con group (Figure 5c).

### 2.4. Fucosterol Increased the Expression of Protein Synthesis-Related Markers in the TA Muscle of Immobilized Mice

**Figure 6:**
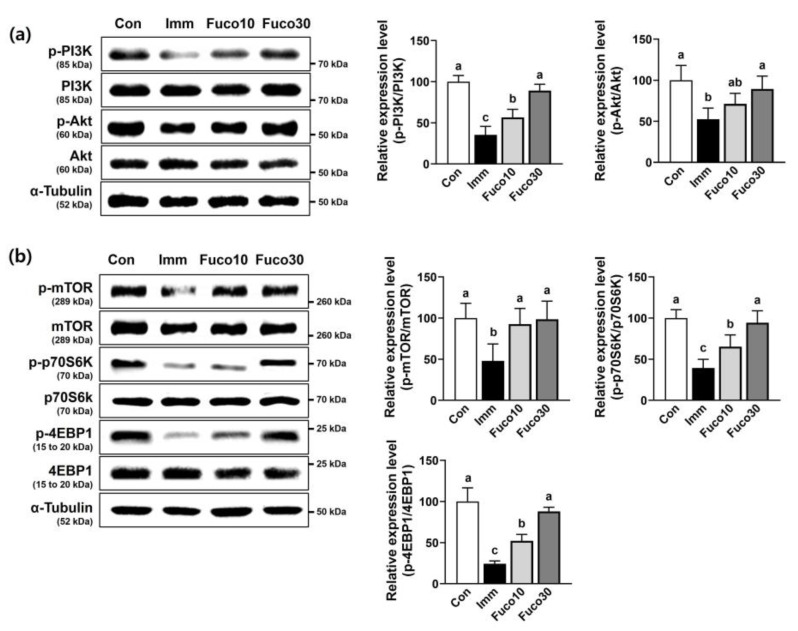
Effect of fucosterol on the PI3K/Akt and mTOR/p70S6K/4EBP1 pathways in TA muscle in immobilized-induced muscle atrophy C57BL/6J mice. After 1 week of muscle atrophy caused by immobilization, mice were subjected to the oral administration of saline or fucosterol (10 or 30 mg/kg/day) for 1 week. (**a**) The protein expression of PI3K and Akt phosphorylation was analyzed in TA muscle by Western blotting. (**b**) The protein expression of mTOR, p70S6K, and 4EBP1 phosphorylation was analyzed in TA muscle by Western blotting. *n* = 3–5 per group. α-Tubulin was utilized as the internal control in Western blot analysis. The data displayed represent the mean values accompanied by the standard deviation. Groups denoted by distinct lettering exhibit statistically significant differences from each other (*p* < 0.05).

Reduced protein synthesis is recognized as a contributing factor in the reduction in muscle mass observed in response to immobilization [17]. The levels of PI3K and Akt phosphorylation, which are essential regulators of protein synthesis, were significantly reduced in immobilized TA muscles. However, fucosterol significantly restored the levels of phosphorylated PI3K and Akt, to a similar level as that of the Con group (Figure 6a). Furthermore, the PI3K/Akt signaling pathway is known to play a crucial role in activating the mTOR/p70S6K/4EBP1 protein synthesis pathway. The immobilization-induced TA muscle of the Imm group significantly reduced the protein expression levels of the phosphorylation of mTOR, p70S6K, and 4EBP1. This reduction was attenuated to the level of the Con group by fucosterol administration (Figure 6b).

## 3. Discussion

Since inflammation is considered a pivotal contributor to the occurrence and development of skeletal muscle atrophy-related diseases, natural compounds with anti-inflammatory properties represent an attractive resource for preventing and treating these diseases [18]. Studies have shown that fucosterol, a characteristic sterol present in algae and seaweed, exerts inhibitory effects in inflammatory cytokine secretion and oxidative stress in macrophages [8]. Therefore, the anti-inflammatory and antioxidant properties of fucosterol can lead to protective effects against skeletal muscle atrophy. In the current study, we first examined the alleviating effects of fucosterol on TNF-α-induced muscle atrophy in C2C12 myotubes and immobilization-induced hindlimb muscle atrophy in C57BL/6J mice. Also, we found that these anti-muscle atrophy effects of fucosterol in C2C12 myotubes and mice were modulated by the Akt/mTOR/FoxO3α signaling pathway.

Skeletal muscle atrophy is developed through an imbalance between the catabolic process of protein degradation and the anabolic process of synthesis [19]. Muscle-specific E3 ubiquitin ligases, including atrogin-1 and MuRF1, play an essential role, responsible for protein degradation, which is activated by the dephosphorylation of FoxO3α [2]. In this study, fucosterol significantly reduced the increase in TNF-α-stimulated atrogen-1 and MuRF1 gene expression in C2C12 myotubes, and these results were also consistent with immobilization-induced TA muscle in mice. Furthermore, the reduction in atrogen-1 and MuRF1 gene expression caused by fucosterol was mediated through the increased phosphorylation of FoxO3α, providing direct evidence for the anti-muscle atrophy effect of fucosterol accompanied with an increase in skeletal muscle mass, volume, and myofiber CSA. Thus, the anti-muscle atrophy effect of fucosterol through the inhibition of FoxO3α activation demonstrates that fucosterol is a potential natural component for preventing the development of skeletal muscle atrophy.

During skeletal muscle atrophy, the Akt/mTOR signaling pathway is inhibited, whereas FoxO3α is activated [2]. Akt, a target protein of PI3K, stimulates mTOR activation, leading to the hyperphosphorylation of both 4EBP1 and p70S6K, thereby promoting protein synthesis [20]. On the other hand, the activation of FoxO3a, an essential regulator of protein degradation, was inhibited by Akt [2]. Thus, the signaling pathway of Akt/mTOR/FoxO3α represents a pivotal mechanism in the mitigation of muscle atrophy by enhancing protein synthesis and concurrently suppressing protein degradation. In the current study, we found that TNF-α treatment in C2C12 myotubes and immobilization in mice significantly suppressed the phosphorylation of Akt, PI3K, mTOR, p70S6K, and 4EBP1. However, fucosterol restored the reduced phosphorylation of Akt/PI3K and mTOR/p70S6K/4EBP1 and prevented atrogin-1/MuRF1 expression by inhibiting FoxO3a. Also, since Akt is known to co-regulate mTOR and FoxO3a, it is likely that fucosterol simultaneously modulates the Akt/mTOR and Akt/FoxO3a pathways. Thus, these results suggest that fucosterol at least works as a dual regulator to stimulate muscle protein anabolism and reduce muscle protein catabolism by activating one target, Akt. The preventive effects of fucosterol in skeletal muscle atrophy can be mediated by the Akt/mTOR/FoxO3α signaling pathway, which likely contributed to an increase in skeletal muscle mass and function by fucosterol administration in the immobilized mice.

The hormone insulin-like growth factor-1 (IGF-1) is recognized as a pivotal anabolic growth factor that stimulates the activation of the PI3K/Akt signaling pathway upon binding to its respective receptor, thereby controlling the process of muscle growth [2]. Studies have demonstrated that the PI3K/Akt signaling pathway is closely associated with skeletal muscle atrophy [21,22]. IGF-1 receptor knockout mice showed a reduction in the PI3K/Akt pathway, which leads to defective skeletal muscle development accompanied with a reduction in the number and area of myofibers [21]. Also, 20-hydroxyecdysone is a plant-derived ecdysteroid hormone known to improve skeletal muscle mass and size. The oral administration of 50 mg/kg 20-hydroxyecdysone for 28 days in male Sprague Dawley rats increased grip strength and stimulated protein synthesis, which was mediated by the activation of the PI3K/Akt signaling pathway [22]. In this study, fucosterol prevented a TNF-α-induced reduction in PI3K and Akt phosphorylation in C2C12 myotubes. Continuously, the TA muscles of immobilized mice administered with fucosterol showed an increase in PI3K and Akt phosphorylation, which contributed to improved grip strength, volume, and mass in hindlimb-immobilized mice. Thus, the anti-muscle atrophy effect of fucosterol via the PI3K/Akt signaling pathway likely contributed to alleviating the immobilization-induced decline in muscle mass, growth, and function. However, a future study is warranted to explore whether fucosterol plays a direct role in inhibiting muscle atrophy-related symptoms by activating the PI3K/Akt signaling pathway using siRNA targeting the PI3K/Akt/mTOR signaling pathway.

Immobilization is a type of muscle atrophy known as disuse atrophy, which can occur in various situations, such as spinal cord injury, prolonged bed rest, confinement in intensive care units, or exposure to microgravity [23]. This condition leads to a significant reduction in muscle mass, fiber size, and the overall number of muscle fibers, ultimately resulting in the wasting of skeletal muscle, which involves an increase in inflammation and oxidative stress, as well as the stimulation of protein degradation [24,25]. Therefore, this study was primarily designed to investigate the effects of fucosterol on immobilization-induced muscle atrophy, and a control group treated with fucosterol under normal conditions was not included. Fucosterol administration significantly attenuated immobilization-induced muscle atrophy, as evidenced by an enhancement in muscle strength with a concomitant increase in muscle volume, mass, and myofiber CSA without body weight changes in the TA muscle of mice. Also, despite the body weight differences observed between the control and immobilization groups due to exposure to immobilization stress, there is no significant difference in body weight between the fucosterol administration group and the immobilization group. These results suggest that fucosterol exhibits an anti-muscle atrophy effect without any toxicological side effects. Since studies have demonstrated that fucosterol does not show toxic effects in animals at doses ranging from 10 to 100 mg/kg/day [26], additional toxicological parameters were not measured in this study. However, further research on the control group treated with fucosterol is necessary to evaluate its broader effects on muscle function and ensure its safety.

TNF-α is known to be a major cause of muscle wasting, as it inhibits protein synthesis and promotes protein breakdown in skeletal muscle [3]. Studies have revealed that TNF-α is a key player in the development of skeletal muscle atrophy, both in vitro and in vivo [4,16,27]. In differentiated L6 and C2C12 myotubes, TNF-α treatment clearly induced muscle atrophy, as evidenced by a decrease in myotube diameter along with an imbalance in muscle proteins [4,27]. Also, mice that underwent muscle atrophy due to immobilization stress exhibited elevated TNF-α expression in the muscles and increased TNF-α levels in the bloodstream [16]. In this study, TNF-α treatment significantly induced muscle atrophy in C2C12 myotubes through an increase in the ubiquitin–proteasome system and a decrease in the protein synthesis pathway. However, this effect was significantly ameliorated by fucosterol. Consistently, in the TA muscle of mice, immobilization stress increased TNF-α and IL-6 gene expression, along with increased atrogin-1 and MuRF1 expression, which was attenuated by fucosterol administration. These results suggest that the preventive effect of fucosterol on skeletal muscle atrophy is attributed, at least in part, to a reduction in pro-inflammatory cytokines in TNF-α-treated C2C12 myotubes and immobilized-induced mice. Consistent with our findings, the strong anti-inflammatory effects of fucosterol have been well supported in studies. Fucosterol significantly inhibited lipopolysaccharide-induced pro-inflammatory cytokine expression and the production of nitric oxide through the inactivation of the NF-κB and p38 MAPK signaling pathways in macrophages [8,9,10]. Also, fucosterol significantly prevented particulate-matter-induced inflammation through the NF-κB/MAPK pathways and Nrf2/HO-1 involvement in macrophages [28]. Thus, our results indicate that fucosterol reduced the expression of pro-inflammatory cytokines, which is a major contributor to the preventive effect of fucosterol on skeletal muscle atrophy.

## 4. Materials and Methods

### 4.1. Chemical Reagents

Fucosterol was bought from Aktin Chemical Inc. (Chengdu, China). Dulbecco’s modified Eagle medium (DMEM, high glucose), fetal bovine serum (FBS), and penicillin–streptomycin solution were acquired from Hyclone (Logan, UT, USA). Horse serum was purchased from Gibco (Gaithersburg, MD, USA). Recombinant rat TNF-α was obtained from PeproTech (Rocky Hill, NJ, USA). Antibodies against phosphorylated (p-PI3K), PI3K, phosphorylated Akt (p-Akt), Akt, phosphorylated mTOR (p-mTOR), mTOR, phosphorylated p70S6K (p-p70S6K), p70S6K, phosphorylated 4EBP1 (p-4EBP1), 4EBP1, phosphorylated FoxO3α (p-FoxO3α), FoxO3α, and α-tubulin were supplied by Cell Signaling Technology (Beverly, MA, USA).

### 4.2. Cell Culture and Differentiation

C2C12 cells, a mouse skeletal muscle cell line, were acquired from the American Type Culture Collection (ATCC, Manassas, VA, USA) and cultured in high-glucose DMEM supplemented with 10% FBS and antibiotics (100 U/mL penicillin A and 100 μg/mL streptomycin) in an atmosphere of 5% CO_2_ at 37 °C. Once the cells reached about 70–80% confluence, the growth medium was replaced with a differentiation medium. The differentiation medium consisted of high-glucose DMEM mixed with 2% horse serum and antibiotics to induce myotube differentiation. The differentiation process continued for 6 days, and the differentiation medium was refreshed every 2 days. After 6 days, fully differentiated C2C12 myotubes were treated with 50 ng/mL TNF-α and fucosterol at 1 or 5 μM for 12 h.

### 4.3. Reverse Transcription–Polymerase Chain Reaction (RT-PCR)

Total RNA was extracted from C2C12 myotubes and TA muscles using TRIzol^®^ reagent (Takara Bio, Otsu, Japan) following the manufacturer’s protocol. Subsequently, cDNA synthesis and PCR amplification were carried out using the GeneAmp^®^ PCR system 2700 (Applied Biosystems, Foster City, CA, USA) with Reverse Transcriptase Premix (Elpis-Biotech, Daejeon, Republic of Korea) and PCR premix (ELPIS-Biotech), as previously described [25]. After this, the PCR products were electrophoresed on a 1.5% agarose gel, stained with Loading STAR Dye (Dyne Bio, Seongnam, Republic of Korea), and visualized using the G:BOX EF system (Syngene, Cambridge, UK)

### 4.4. Western Blot Analysis

The molecular mechanism of the Akt/mTOR/FoxO3α signaling pathway was confirmed through Western blot analysis in both C2C12 myotubes and TA muscles, as previously described [29]. The visualization of protein expression was achieved using the G:BOX image analysis system and Genesys software version 1.3.9.0 (Syngene).

### 4.5. Animal Experiment

Male C57BL/6J mice at the age of 7 weeks (Dae Han Biolink Co., Ltd., Umsung, Republic of Korea) were housed under controlled conditions (25 ± 2 °C temperature, 55 ± 5% relative humidity, and 12 h light–dark cycles) with ad libitum access to tap water and food during the experiment period. After 1 week of circulation, the mice were divided into 4 groups (*n* = 7 per group) as follows: (i) Con (control); (ii) Imm (immobilization-induced skeletal muscle atrophy group); (iii) Fuco10 (immobilization and fucosterol 10 mg/kg/day-treated group); and (iv) Fuco30 (immobilization and fucosterol 30 mg/kg/day-treated group). Skeletal muscle atrophy was induced by immobilization stress to the right hindlimb for 1 week, while the left hindlimb served as a non-immobilized control, as previously described [16]. While the mice were under anesthesia with 350 mg/kg of 2,2,2-tribromoethanol (Sigma-Aldrich, St. Louis, MO, USA), one tine of a surgical staple was inserted near the toe on the plantar side of the foot, while the other tine was inserted into the distal portion of the GA muscle by using an Autosuture Royal 35W skin stapler (Unidus, Seoul, Republic of Korea). After 1 week of muscle atrophy caused by immobilization, the staple was removed while under anesthesia. The mice in the fucosterol treatment groups, Fuco10 and Fuco30, received the oral administration of fucosterol for 1 week, while the mice in the Con and Imm groups were orally administered with saline. At the end of the treatment period, grip strength and muscle volumes were measured. Then, the mice were sacrificed by cardiac puncture under anesthesia. The GA, SOL, TA, and EDL muscles were harvested and snap-frozen in liquid nitrogen for gene and protein expression analysis or fixed in 10% formalin for histological analysis. All animal procedures were approved by the Institutional Animal Care and Use Committee of Yonsei University (Permit No., 201612-519-02; Approval Date, 31 January 2017).

### 4.6. Micro-CT Imaging

Micro-CT imaging was employed to quantify muscle volume utilizing an animal positron emission tomography/CT/single-photon emission tomography system (Siemens Inveon, Knoxville, TN, USA) at the Center for Evaluation of Biomaterials (Pohang Technopark, Pohang, Republic of Korea).

### 4.7. Grip Strength Test

A grip strength test was conducted on mice using a Chatillon force measurement tool (Columbus Instrument, Columbus, OH, USA), as previously described, to determine the forelimb/hindlimb grip strength of mice [29]. The combined forelimb and hindlimb grip strength (4 paws), as well as the forelimb grip strength (2 paws), were measured at the end of the oral administration period. Each mouse was permitted to grasp the bar, while its tail was gradually retracted until the mouse released the bar. A total of five consecutive trials were conducted for each mouse to ascertain the peak value.

### 4.8. Histological Analysis

TA muscle tissues were fixed with 10% formalin solution, stained with H&E, and examined under an Eclipse TE2000U inverted microscope with twin charged-coupled device cameras (magnification, ×200; Nikon, Tokyo, Japan). One block of TA tissue was obtained from each mouse, and two random fields per block were analyzed for image analysis. The CSA of each myofiber was quantified using ImageJ software (version 1.50b; National Institutes of Health, Bethesda, MD, USA). Representative images of the quantified CSA are represented.

### 4.9. Statistical Analysis

Data are shown as the mean ± standard deviation. Group differences were examined using two-way factorial analysis of variance and considered significant at a probability level of *p* < 0.05. All statistical tests were followed by Duncan’s multiple comparison tests conducted with SPSS 23.0 (IBM, Armonk, NY, USA).

## 5. Conclusions

In summary, the present study examined whether fucosterol attenuated skeletal muscle atrophy in both C2C12 myotubes treated with TNF-α and the immobilized hindlimb muscles of C57BL/6J mice. Fucosterol reversed the immobilization-induced decrease in muscle strength by recovering the muscle volume, mass, and myofiber CSA. At the molecular level, fucosterol inhibited atrogin-1 and MuRF1, while stimulating the phosphorylation of both 4EBP1 and p70S6K, along with a reduction in TNF-α and IL-6 gene expression by modulating the Akt/mTOR/FoxO3α pathway in vitro and in vivo. Consequently, if the preventive effect of fucosterol on skeletal muscle atrophy is shown in clinical tests, then fucosterol could be used as a potential marine agent in preventing muscle atrophy and improving muscle function.

## Data Availability

The data presented in this study are available on request from the corresponding author.
